# The role of fear in mental health service users’ experiences: a qualitative exploration

**DOI:** 10.1007/s00127-015-1028-z

**Published:** 2015-02-22

**Authors:** Angela Sweeney, Steve Gillard, Til Wykes, Diana Rose

**Affiliations:** 1 Population Health Research Institute, St George’s University of London, Cranmer Terrace, London, SW17 0RE UK; 2 Department of Psychology, Institute of Psychiatry, Psychology and Neuroscience, King’s College London, London, UK; 3 Department of Health Service and Population Research, Institute of Psychiatry, Psychology and Neuroscience, King’s College London, London, UK

**Keywords:** Fear, Control, Stigma, Qualitative research, Service users’ experiences

## Abstract

**Purpose:**

Although studies suggest that fear plays an important role in shaping mental health service users’ experiences, evidence is patchy and the contexts, conditions and consequences of fear have rarely been researched. This paper explores the role of fear in adult mental health service users’ lives and describes its implications for mental health services.

**Methods:**

Four community health service user focus groups (N32) were held. Each group was reconvened after 7–14 days. An initial thematic analysis generated a service user definition of continuity of care (reported elsewhere). A Straussian ‘secondary grounded theory analysis’ was conducted to gain a deeper understanding of participants’ experiences.

**Results:**

‘Being afraid’ was identified as a core process, with power and control, and stigma and discrimination found to have explanatory power in determining how and why fear manifests. Consequences included distrusting staff, cooperating reluctantly, learning reticence, delaying help-seeking, avoiding services, feeling unsafe in the community and avoiding exposure as a service user.

**Conclusions:**

Our model suggests that fear plays a substantial role in the lives of adult mental health service users. This has particular consequences for therapeutic relationships, engagement with services and engagement with the wider community. This lack of engagement is associated with adverse outcomes. Further research into the role of fear and the factors that mediate against it is warranted.

## Introduction

Evidence suggests that fear plays an important role in shaping the experiences of people who use psychiatric services [1, 2]. Yet the conditions, causes and consequences of fear have rarely been researched [3], despite calls for a sociology of fear that “must examine the cultural matrix within which fear is realised and attend to the patterns of social activity routinely associated with it” [4].

Fear and mental health have been linked in a number of ways. It has been argued that fear drives the contemporary mental health system [5]. Mental health policy revolves between promoting civil liberties and legally constraining freedoms, with ‘safety’ currently emphasised over ‘support’ [6]. Contemporary mental health services have become increasingly risk averse, most notably through the introduction of Community Treatment Orders or Mandated Community Treatment in Europe, North America and Australasia. It has been claimed that such coercive practices, fuelled by stigma and fear of ‘dangerous’ service users, prevent people from accessing support [5]. For example, studies have identified fear of coercive treatment as a barrier to help-seeking [7, 8].

Fear has also been conceptualised as a causal factor in mental distress. In the UK, it has been claimed that people are becoming more fearful, and this is impacting on our collective experiences of mental distress [9]. These collective fears are fuelled by the socio-historical context in which we live, including our institutions, dominant culture and social and material relations [9].

There is patchy evidence that fear operates differently for different social groups and in particular for Black and Minority Ethnic service users. Suicide and homicide inquiries have repeatedly linked tragedies to institutional racism, stereotyping and perceptions of dangerousness [10]. The *Breaking the Circles of Fear* study explored the experiences of Black service users, their families and professionals through analysing the harmful and pervasive role of fear, informed by explorations of power, control, stigma and discrimination [1]. The premise of the study was that there are many layers of fear and that if you combine these different layers of fear—fear of Black people, fear of mental illness and fear of mental health services—you arrive at a pernicious circle of fear, a circle that impacts negatively on the engagement of Black people with services and vice versa,

A number of studies have found that distinct social groups experience fear differently according to their social positions, roles and relations. For example, mothers experiencing psychosis have particular fears about the effect of their distress on their children and the potentially intrusive role of social services [11]. Interviews with adolescents diagnosed with depression found that ‘“living in the shadow of fear” emerged as the essence of the adolescents’ experiences and ultimately defined what it was like to live with depression’ [12].

Experiential research has demonstrated that being given a mental health diagnosis forced service users to confront the fear and stigma of mental distress, negatively impacting on help-seeking [13]. However, whilst stigma is feared, it is not always experienced or negative [14]. Interviews with Black and Minority Ethnic women who experience mental distress identified fears around diagnoses which were so strong as to damage recovery [15]. Service user research has also found that fear plays a key role in shaping experiences and avoidance of services in people considered ‘hard to engage’ [2].

One consequence is that service users fear disclosure of mental health problems even to their GPs because they fear losing control, external judgement, treatment, losing one’s children, and being institutionalised.

This is a patchwork picture built from disparate research studies. To date there has been very little research into the conditions, causes and consequences of fear, and an overarching conceptual model of the role of fear is needed. In this paper, we develop and describe an empirically grounded conceptual model of the role of fear in the lives of adult mental health service users and describe some consequences including impact on engagement with mental health services.

## Materials and methods

This is a ‘secondary grounded theory analysis’ of data collected in a study that explored service users’ definitions of, perspectives on and experiences of continuity of care [16–18]. Thirty-two mental health service users were recruited from day centres, service user groups and community mental health teams (CMHTs) in two south London NHS trusts. Sites were visited to explain the research and discuss participation. Where sites agreed to participate, day centres and user group meetings were attended and the research explained to interested service users. CMHT staff passed information sheets to service users who then contacted the researcher directly to discuss their participation. The main socio-demographic characteristics of the participants are shown in Table 1.

**Table 1:** Participant demographic characteristics (*N* = 32)

Age (mean years)	47 years
Gender (female:male)	37.5:62.5 % (12:20)
Ethnicity	
White British, Irish or other	75 % (24)
Asian/Asian British or Chinese	6 % (2)
Black/Black British	6 % (2)
Mixed heritage	12.5 % (4)
Length of contact with services (mean)	16

Four focus groups facilitated by service user researchers were held and each group was reconvened seven to 14 days later. Groups had 4–12 participants, lasted approximately 120 min and were held in comfortable and familiar settings. Written informed consent was given prior to participation. Initial groups began with participants telling their stories of first contact with mental health services. Participants then discussed key areas based on a topic guide which focused on relationships with key staff members, including what did and did not work well and continuity of contact, and support services, including how services fit together, support needs in a crisis and gaps in care. Groups were audio-recorded and transcribed by an independent transcriber. Initial groups were analysed thematically [19]. Repeated groups began with member checking [20] through a detailed discussion of the interim thematic analysis, before focusing more closely on the concept of continuity of care. The study received ethics committee approval (Wandsworth NHS Research Ethics Committee reference 01.42.8).

## Secondary analysis

The first phase was a microscopic analysis of the entire dataset using a combination of open and axial coding [16]. Individual words, phrases, sentences, paragraphs, and non-verbal transcribed information were examined. Through making constant comparisons and asking questions about the data (the basic procedures of Straussian grounded theory), ideas, emerging concepts, patterns, differences and contradictions both within and across participants and transcripts were identified and systematically recorded in memos. This generated a coding frame that was applied to all transcripts using qualitative data analysis software (winMAX 98).

The second phase of the analysis explored those codes for the properties and dimensions of categories—concepts, actions and processes—that might have explanatory power in understanding what was shaping participants’ experiences. Visual grids were produced to develop categories; each grid containing category labels, examples from the data, speaker, group, location in the transcript and frequency. Grids were then used to write analytic stories summarising how the category emerged, the implications for findings and inter-relationships between categories. A single core category was identified—fear—that appeared to have explanatory and predictive power across participants’ experiences. A small number of explanatory sub-categories—power, control, stigma and discrimination—elucidated how and why the core category of fear operated (i.e. the conditions which mediated experience of fear) in a number of specified contexts. This represented an emerging model of the data.

In the final phase of the analysis the emerging model was validated against the raw data to test its fit and uncover additional elements. A coding frame was generated from the core category and sub-categories and applied to the data using a qualitative software package (MAXqda 2) to facilitate systematic and intensive comparison of the conceptual model with the data. Negative instances (data that contradicted the emerging model) were considered to explore how the model accounted for variation and deviant cases [21]. All analyses were conducted by AS, a service user researcher, in discussion with DR, a service user researcher, and TW, a clinical academic.

## Results

The core category that emerged from the microscopic analysis was fear or ‘being afraid’. Fear was expressed in every focus group without probing, indicating that it had wide relevance, and was often central to what was occurring in the data, with other categories related to it. One participant neatly encapsulated the notion that fear resonates with most service users:

“Most people that have mental health problems, and I’m just half-guessing this but it seems to me that the one big problem that’s, that runs through all our cases is the fact that we’re quite scared, especially at first when we don’t know what’s happening.” (White male)

The microscopic analysis suggested that fear operated in three main contexts: psychosis, services and community. This has strong resonance with the layers of fear identified in the *Breaking the Circles of Fear* study [1]. Within each context, two major sub-categories (sets of conditions) had explanatory and predictive power in determining how, when and why fear was experienced: power/control and stigma/discrimination. As the analysis proceeded, a further sub-category—climate of fear—added explanatory power to the model. The resulting model is illustrated as a grid in Table 2 and presented in the analytic story that follows.

**Table 2:** A conceptual model of fear indicating inter-related sub-categories (conditions as column and context as row headers, respectively) that comprise the core category of ‘being afraid’, and the analytical codes used to develop sub-categories

Core category: being afraid	Sub-categories (conditions)			
	Power and control	Stigma and discrimination	Climate of fear	Negative instances
Sub-categories (contexts)				
Psychosis	Fear worse at certain times Fear causing illness/symptoms Fear linked to illness/symptoms/relapse Experience/education decreasing fear Fear when first ill	Treated as an illness From user to user From being ill/diagnosed	Funding cuts No action until very ill Users: no rights/disempowered Not being believed Staff are gatekeepers Feeling/being at risk Suspicious of staff/services Lack of respect/‘them and us’ Lack of trust Fear of reprisals/threats/intimidation Unable to be/express yourself	Knowledge decreasing fear Not treated as an illness Positives of the illness
Services	Fear of dependency Fear linked to staff change Fear of compulsion	Information and stigma Staff stereotyping/discriminating Fear of treatments/services		Able to be/express yourself Compulsion = safety Services/treatment = safety Respected by staff Trust staff Direct payments
Community	Fear connected with life event Fear of police/arrest	Community stigma and fear Feeling unsafe in the community	Unable to be/express yourself Government proposals Fear of community rejection/reaction Negative media images	Positive media images Positive community Positive government proposals Positive police experiences
				Challenges/contradictions to the theory
Other			Fear all pervading Frightened to talk	

### Fear, power and control

#### Psychosis

The most common context of fear was the experience of psychosis. One participant described mental distress as “our fears” and psychosis as “scary” and “very, very frightening”. Others believed someone was trying to kill them or their loved ones, or felt unable to distinguish between reality and non-reality. These experiences affected people’s confidence, relationships, and ability to travel or be in public spaces.

The worst thing about my state of health is fearing I’m missing out on life (White male).

Fear was particularly acute when first experiencing psychosis as people did not understand what was happening and lacked control over their experiences.

I think it dragged on so long because I was probably so scared and because I didn’t have much knowledge about what it meant to have a diagnosis of manic depression (White female).

Fear is compounded where people believe they may never recover, or have a background fear of crisis. These fears link to power and control: the experiences of psychoses are unknown, their return unpredictable and their impact immense. Over time, some people learnt more about their experiences and vulnerabilities to crisis, reducing their fear (i.e. where a condition ameliorated experience of fear—in this case increased control—this positive experience was also informative of the model).

As you get older I feel you become more aware, the experiences are not so frightening, I mean they are frightening but they, because you know what to expect you know how to deal with them (White male).

Although experiences of psychosis were associated with being afraid, some people stressed positives such as increased enlightenment and tolerance. However, these views were not widely shared.

#### Services

A small number of participants were concerned that they would be subject to compulsory treatment or detention. Some who had experienced compulsion tried to avoid services altogether. One participant described needing someone to talk to during crises, yet feared being given medication. Consequently, he did not contact services: “I don’t want to be in a situation where I can be forced”. Another participant co-operated with treatment through fear of forced detention:

*Participant* (White male): they’ve still got some sort of power over you and it’s as if they’re sort of, you know, I feel as though, well I just feel I’ve got to go along with what they say, whether you agree with it or not as a human being, you know, and you should have rights, certain rights.

*Facilitator*: Why do you feel that you’ve got to go along with it?

*Participant*: Because I don’t want the threat of going back into the hospital.

Service gaps were described, such as post-hospitalisation support and supportive listening. Consequently, some people were afraid that what was needed would not be provided, whilst unwanted services would be forced.

Compulsion was not simply viewed with fear. One participant described how being sectioned following attempted suicide saved his life. However, he saw services as important for emergency intervention but little else. Services were sometimes described in terms of safety and security. Rapid access to support, such as emergency psychiatric clinics or crisis hotlines, made some feel safer.

It feels great. It makes me—to know that there is a network there which will hold me, which won’t let me fall to the ground; you know, they are there to catch me before I drop completely, it’s a very comforting feeling (White female).

This person was firmly in control of her emergency care, preventing the fear that arises when accessing support is beyond one’s control. However, it was atypical, with other participants considering her ‘privileged’ and ‘lucky’. Indeed, access to services was consistently described negatively: staff have the power to grant or deny access and their decisions are often experienced as arbitrary and therefore frightening.

Participants described not being believed by staff, particularly regarding experiences of psychosis, deterioration and medication side-effects. One participant explained that rather than asking psychiatrists for help and receiving it, “You’ve got to persuade him what you want”. The combination of not being believed and finding access problematic occasionally had serious consequences, including being denied access to services, not wanting to seek help, and physical and mental deterioration.

I said, I don’t feel well again, I feel as though I need to come back into hospital, and he turned round and said to me, “What’s this? You don’t think this is a holiday camp?” (White male).

#### Community

There were two obvious instances where power and control contributed to service users’ fears in the context of community. First, one participant with a dependent family experienced redundancy. This loss of control over his life led to deep feelings of fear and insecurity, leading to a breakdown and 3 years in and out of hospital. The second participant felt he could be arrested and hospitalised without committing a crime (explored under a climate of fear in community).

### Fear, stigma and discrimination

#### Psychosis

Fear around psychosis sometimes extended to a fear of stigma and labelling. One participant described his partner viewing his actions and emotions through the lens of his diagnosis, making it difficult to display normal human emotions.

Just to be known to have a mental health problem and then to have an argument with somebody can be … seen as you having some psychotic episode (Black male).

Conversely, staff who did not treat people as an illness were described favourably.

I got a new psychiatrist who was absolutely brilliant … he didn’t talk to me as if I was somebody with an illness; he talked to me as if I was a person (White female).

Stigma emanated not only from staff and family: one participant described his fear of service users making him cautious about attending a day centre. However, another participant could express herself to peers in a way that she could not with others for fear of rejection.

#### Services

Fear in the services context was sometimes caused by discrimination.

They meet you and they judge you, they stereotype. We all do it, but in that kind of environment it’s detrimental, you know (South European male).

This is particularly damaging as staff have the power to enforce or withdraw treatment. The above participant felt that dual diagnosis service users “are looked at differently and discriminated definitely, I have no doubts about it”. He described staff witnessing his self-harm contemptuously. Another participant believed that as a large Black male, staff perceived him as a threat and treated him accordingly. He recounted seeing a small white man acting aggressively without consequence, “if that had been me, they would have given me medication—and pinned me down”.

However, another participant believed that 30 years ago services were ‘frightening’ but now mental health is more understood. For another participant, in-patient stays provided a respite from community stigma. Thus, services can be both a source of and refuge from stigma and discrimination.

#### Community

A number of participants described societal stigma and discrimination, one believing this causes a fear of service users, “the general public—you know, they fear us somehow”. Another participant felt that discrimination, race and mental health were entangled:

Especially being a Black male, I feel emotional just talking about it. You know, the –stereotype of a mad, Black man.

Similarly, a further participant described discrimination on the basis of her immigrant status. This made her feel unsafe in the community, exacerbated her mental distress and increased her reliance on services. Public education was sometimes recommended to tackle discrimination, with one participant educating the police about race and mental health. This demonstrates the positive strategies that service users believe could tackle discrimination and related fears.

### Climate of fear

#### Services

There was evidence that insidious feelings, states, actions, interactions and consequences create a climate of fear. For example, service users experience staff power over them as infantilizing, with fears that not doing as one is told could lead to compulsory treatment: “you have to do as you’re told and it’s, whether you like it or not”. Some participants described being ignored, laughed at, humiliated, belittled and patronised.

Participant 1 (White male): I find them patronising and they—

Participant 2 (White female):—treat you like a child!

Others expressed deep suspicions: staff want to “keep you back”, “down there”, or you are simply “a number”. There was also some evidence that resistance could have negative outcomes.

Of course, as soon as you start arguing with a psychiatrist, you must be unwell (White male).

Some people altered their behaviour as a result, learning reticence and hiding their emotions.

They are quick to make judgements and make decisions that you might not agree with so you start to learn what are the things that you should avoid to tell them because it might influence their attitude (White male).

One participant felt disempowered by being unable to express the full range of human emotions.

You can’t live your life; you can’t be happy one minute and sad the next, angry the next, happy the next – the whole range of emotions that we want to feel as human beings. That thing’s been taken away from us (Black male).

However, he was able to express himself to two support workers in hospital without fear of reprisal. Similarly, another participant valued a psychiatric nurse with whom she could discuss her fears.

#### Community

There was evidence of a climate of fear operating within communities. Some participants feared community rejection, with one participant losing her friends following a breakdown. This fear led some to hide their emotions, fear the loss of asylum, present the “façade” of normality or hide their service use:

I’ve even asked the ambulance in the past, and they’ve picked me up at the end of the road (White female).

Many described loneliness as their biggest problem. Isolation was exacerbated by media representations of dangerous service users. One participant even felt that the media had played a role in her friend’s diagnosis of her:

I had a friend who said I was a psychopath because I had a personality disorder, and that frightened me to bits! I said, “Gosh, I’ve never wanted to harm anyone!” She must have heard it on some TV programme, you know (White female).

Interactions with the community were not all fuelled by fear, with positive interactions described with the police, family, friends and neighbours.

I tried to commit suicide. And if it wasn’t for my neighbour’s attention at the time, I wouldn’t be telling the story today (White male).

Other service users were also an important source of support, offering understanding and friendship.

## Discussion

Our findings signify the substantial and pervasive role of fear in the lives of adult mental health service users. Whilst our conceptual model of fear has been presented as arising in three distinct contexts (psychosis, services and community) with three distinct explanatory conditions (power and control, stigma and discrimination and climate of fear), the links between the conditions and consequences of being afraid are inevitably multiple and complex. For example, discrimination requires one group to have the power to stigmatise and discriminate against another [22]. Additionally, a climate of fear can arise where an imbalance of power and control is exercised alongside stigma and discrimination. Despite the clear significance of fear, there is little literature that directly explores its impact on service users. This discussion will explore the consequences of experiencing fear in relation to a wider mental health literature.

### Fear and loss of control

In the context of psychosis, participants repeatedly linked the loss of control surrounding psychosis to fear. The finding that psychosis—and resulting hospitalisation—is traumatic is not new [23]. That experience of fear was associated for our participants with issues of power and control across multiple contexts was a further echo of the findings reported in *Breaking the Circles of Fear*:

Concerns about the unpredictable nature of ‘the illness’, loss of control and the overall impact on their quality of life were further sources of fear for service users. [1, p25]

### Fear and engagement with services

Service users are also exposed to a loss of control through the possibility and experience of treatment and detention without their consent. This, alongside experiences of discrimination by powerful staff, had a number of consequences. Service users sometimes distrusted staff, and had a number of strategies for managing their interactions including cooperating reluctantly, adjusting behaviour and learning reticence. This contributed to a staff/service user divide and damaged therapeutic engagement [24, 25]. Research has found that approximately one-third of service users report fear of coercion as a barrier to seeking support [7, 8]. Compulsion is particularly feared when services are experienced as harmful, whilst what is wanted is absent; this can be as simple as a listening ear [26]. Davies and colleagues found that service users considered ‘hard to engage’ typically wanted contact with services yet avoided them where they were experienced as intrusive, controlling or over-reliant on medication, and wanted services were unavailable [2]. Similarly, *Breaking the Circles of Fear* concluded:

Paradoxically, Black communities receive the MH [mental health] services they don’t want, but not the ones they do or might want. [27]

Likewise, service users in our study on occasion felt that they needed support and a listening ear, but did not contact mental health services through fear of unwanted compulsory treatments.

As well as being a source of support, studies have found that other service users can be experienced as frightening, particularly on acute wards. Service users in acute settings employ a number of strategies to manage difficult relationships with staff and peers including avoiding other service users, escaping [28, 29], retreating, and learning which staff are to be avoided [30].

### Fear and isolation

In the context of the community, public fear of service users has consistently been described as a key cause of discrimination, and evidence suggests that it is a barrier to seeking support from mental health services [31, 32]. Our participants sometimes feared community rejection: many had lost friends, and described loneliness as their biggest problem. Similarly, an international literature review concluded, ‘rejection and avoidance of people with mental illness appear to be a universal phenomena’ [33]. This can be so severe that it causes mental distress [24], and in our study occasionally resulted in people needing additional support from services. Furthermore, people sometimes felt unsafe in the community, and some participants avoided exposure as a service user, hiding their service use and diagnosis from others [33]. Unsurprisingly, the support of peers was highly valued, often over and above that of staff, family or friends.

Cutting across contexts, our participants sometimes felt unable to be themselves, tried to appear ‘normal’, and felt that their words and actions were interpreted through the lens of their diagnosis. Research has found that people who are compulsorily detained describe being denied the normal range of emotions [34]. This inability to express oneself was experienced as disempowering by some participants, particularly a Black male participant who felt he was viewed as big, Black and dangerous, a common stereotype [35].

## Conclusion

Our participants were often aware that people who experience psychosis can be portrayed as dangerous and a potential threat to the public. Mental health policy has responded to perceptions of dangerousness by facing in two directions at once [5]. There is a focus on risk management and public order, leading to policies of control and compulsion such as the Mental Health Act 2007. Yet policy is also shaped by the human rights agenda and social inclusion, leading to a focus on choice and anti-discrimination [36]. This has meant that service users are encouraged to enact choices whilst fearing compulsion if they do not make the choices that are sanctioned by powerful mental health professionals. Those who choose not to engage with mental health services can be labelled resistant [2]. The consequences of fear identified in our conceptual model shed new light on this discourse by providing some explanation as to why people might choose to avoid mental health services. This could be seen as a rational decision, given the fear, discrimination and powerlessness that can result from service contacts.

Overall, our conceptual model strongly indicates associations between experience of fear and a number of processes that have been shown in a wider literature to mediate outcomes such as hospital admission and compulsory treatment; therapeutic relationship, engagement with services and engagement with the wider community [37–39]. As such this study makes a convincing case for further research into the role played by fear in shaping the experience and use of mental health services.

### Strengths and limitations

In our secondary grounded theory analysis, we employed a number of techniques to increase validity, including coding negative instances and peer debriefing [20, 21]. The theoretical categories of the model were further validated and contextualized in the extant literature, in line with a grounded theory approach [40]. Previous survivor-led research has indicated that people experience fear through their use of mental health services [2, 13, 15]. This study has further demonstrated the potential of survivor-led research in understanding and explaining service users’ experiences [41].

Limitations include that pre-existing data were analysed and theoretical saturation may not have been reached [42]. In particular, fear relating to experiences of power and control within the community is a notably under-developed category, with little supporting data. As this was a secondary analysis, we were unable to add questions to the topic guide, or theoretically sample for participants with experiences relating to this category. Future research should explore power and control in community settings and the ways these interact with fear and mental health. Furthermore, the sample was purposive and self-selecting with the primary aim of ensuring experiences of longitudinal and cross-sectional continuity of care. Fear may operate differently for people who experience psychosis compared to those who do not; for example, there is evidence that people with psychosis experience greater overt discrimination than people who do not experience psychosis [43]. Further primary research should explicitly set out to explore each of the contexts, conditions and consequences as articulated by our model, and explore heterogeneity amongst service users.
